# The Factors Predicting Concordant Epidermal Growth Factor Receptor (EGFR) Mutation Detected in Liquid/Tissue Biopsy and the Related Clinical Outcomes in Patients of Advanced Lung Adenocarcinoma with *EGFR* Mutations

**DOI:** 10.3390/jcm8111758

**Published:** 2019-10-23

**Authors:** Chia-Yu Kuo, Mei-Hsuan Lee, Ming-Ju Tsai, Chih-Jen Yang, Jen-Yu Hung, Inn-Wen Chong

**Affiliations:** 1 Division of Pulmonary and Critical Care Medicine, Department of Internal Medicine, Kaohsiung Medical University Hospital, Kaohsiung 807, Taiwan; goba2356@gmail.com (C.-Y.K.); mhsuan99@gmail.com (M.-H.L.); SiegfriedTsai@gmail.com (M.-J.T.); chjeya@cc.kmu.edu.tw (C.-J.Y.); chong@cc.kmu.edu.tw (I.-W.C.); 2 Graduate Institute of Medicine, College of Medicine, Kaohsiung Medical University, Kaohsiung 807, Taiwan; 3 School of Medicine, College of Medicine, Kaohsiung Medical University, Kaohsiung 807, Taiwan; 4 Department of Respiratory Therapy, College of Medicine, Kaohsiung Medical University, Kaohsiung 80708, Taiwan

**Keywords:** lung cancer, adenocarcinoma, *EGFR*, liquid biopsy

## Abstract

Liquid biopsy to identify epidermal growth factor receptor (*EGFR*) gene mutations from circulating tumor DNA (ctDNA) for lung adenocarcinoma is less invasive than traditional tissue biopsy. Most patients have concordant results in liquid/tissue biopsy, while the clinical significance of concordant results remains unclear. Our study aimed to evaluate the predicting factors and clinical outcomes associated with concordant results in liquid/tissue biopsy in newly diagnosed lung adenocarcinoma patients with *EGFR* mutations. In the 80 patients of stage III or IV lung adenocarcinoma, 51 patients had *EGFR* mutations detected in tissue samples, while 33 (65%) of them had concordant results shown in liquid biopsy. Multivariable regression analysis showed that lymph node involvement (adjusted odds ratio (95% CI): 8.71 (1.88–40.35), *p* = 0.0057) and bone metastasis (adjusted odds ratio (95% CI): 9.65 (1.72–54.05), *p* = 0.0099) were the independent predicting factors for concordant results. Forty of these 51 patients were stage IV and were treated with *EGFR* tyrosine kinase inhibitors (TKIs). The concordant results in liquid/tissue samples were associated with significantly poorer progression-free survival (PFS) in univariate analysis. However, multivariable analysis showed that lymph node involvement was the only independent predicting factor for poorer PFS, while concordant results in liquid/tissue samples were excluded during variable selection. The concordant results in liquid/tissue samples might indicate a larger tumor burden, which actually contributes to poorer PFS.

## 1. Introduction

Lung cancer is the most common cause of cancer-related mortality worldwide [1]. About 80% of all cases are non-small cell lung cancer (NSCLC), among which the most common cell type is adenocarcinoma. More than half of these NSCLC were diagnosed in advanced stage [2]. In the past, the only treatment for advanced NSCLC was platinum-based doublet chemotherapy, resulting in a median overall survival (OS) period of around 8 months [3]. Clinical practice has changed since the development of epidermal growth factor receptor (EGFR) tyrosine kinase inhibitors (TKIs), with the discovery of *EGFR* driver gene mutations in NSCLC. Patients with NSCLC harboring such mutations, such as exon 21 L858R point mutation and exon 19 deletion, have better progression-free survival (PFS) when treated with *EGFR* TKIs [4,5,6]. It is therefore very important to determine the presence of *EGFR* mutation in NSCLC.

Initially, tissue samples, biopsied from either primary tumor or metastatic lesions, had been used for the *EGFR* mutation testing. The procedures of tissue biopsy, including bronchoscopic biopsy, computed tomography-guided biopsy, and surgical biopsy, are all invasive, and bring risks of some complications, such as hemoptysis, pneumothorax, and pneumonitis [7,8,9]. Another limitation of tissue biopsy is the tumor heterogeneity, especially in patients with advanced stages. The results of *EGFR* gene testing might be different in various parts of the cancer, especially in the metastatic sites, so tissue biopsy from one part of a solitary tumor might miss the intra-tumoral and inter-metastatic molecular heterogeneity [10,11]. Furthermore, malignant cells could be found in 72.9% of specimens under pathological examination [12]. Repeated biopsies may sometimes be required to obtain sufficient cancer tissue for gene testing [13].

Liquid biopsy, identifying the genotype of tumor cells from circulating tumor DNA (ctDNA) of the patients’ blood, is a less invasive method. Since the report by Mandel and Metais in 1948, fragmented DNA in the cell-free component of serum has been a field of active research [11]. Investigation of cell-free DNA has been conducted in many disciplines, such as exercise, end-stage renal failure, stroke, myocardial infarction, surgery, and trauma [14,15,16,17]. In the oncological field, studies of cell-free DNA derived from tumors, also known as ctDNA, has dramatically increased recently, mainly because of the development of the genomic technologies that allow detection of rare gene mutant variants of DNA [11]. In recent studies of NSCLC, liquid biopsy was shown to detect driver gene mutations, such as *EGFR* and anaplastic lymphoma kinase (ALK) mutation, and tumor mutational burden [18,19]. Compared to tissue biopsy, liquid biopsy done at disease progression can reduce the necessity of invasive biopsy procedures, the risk of biopsy related complications, and the cost of the complication-related hospitalization [20].

Most patients have concordant results in liquid/tissue biopsy, while a few patients have discordant results in the gene testing of tissue biopsy and liquid biopsy. The factors related to concordant results and the clinical significance of concordant results remain unclear. Our study aimed to evaluate the predicting factors and clinical outcomes associated with concordant results in liquid/tissue biopsy in newly diagnosed lung adenocarcinoma patients with *EGFR* mutations.

## 2. Materials and Methods

### 2.1. Study Population

This retrospective study was conducted in Kaohsiung Medical University Hospital between June 2016 and August 2018. Treatment naïve stage III or IV lung adenocarcinoma patients with *EGFR* mutation tested from both their biopsied tumor tissue and liquid biopsy at their diagnosis were enrolled. All patients received imaging studies, including computed tomography of the chest, brain magnetic resonance imaging (MRI), and whole-body bone scan, to determine the extent of cancer invasion, lymph node involvement, and distant metastasis. The clinical stage was determined according to the American Joint Committee on Cancer (AJCC) Cancer Staging Manual, 7th Edition.

The Institutional Review Board (IRB) of Kaohsiung Medical University Hospital (KMUH) approved this study (KMUHIRB-G(II)-20190024) and waived the need for written informed consent from all patients.

### 2.2. DNA Extraction, Amplification, and Detection

The genomic DNA was extracted from formalin-fixed and paraffin-embedded (FFPE) tumor tissue samples, and *EGFR* mutation was tested by using Qiagen^®^ *EGFR* RGQ PCR kit (QIAGEN, Hilden, Germany) as in our previous studies [21,22,23,24,25,26] or Cobas^®^ *EGFR* Mutation Test v2 (Roche Diagnostics, Rotkreuz, Switzerland) (for the latest 6 samples). The previous validation exam in the Department of Laboratory Medicine, KMUH showed excellent correlation between the results obtained from these two kits. For liquid biopsy, the plasma ctDNA was extracted and then analyzed by Cobas^®^ *EGFR* Mutation Test v2, which was a real-time PCR test for the quantitative detection and identification of mutations in exons 18, 19, 20, and 21 of the *EGFR* gene.

### 2.3. Definitions of Variables

The *EGFR* mutations detected in tissue samples were taken as the reference, to which the mutations detected in plasma ctDNA were compared. The patients with the same *EGFR* mutation patterns detected in both biopsied tissue samples and plasma ctDNA were classified as “concordant” group, whereas those having different mutation patterns in their plasma ctDNA versus their tissue samples were classified as “discordant” group.

Patients with stage IV disease who received first-line *EGFR* TKIs were further extracted for outcome analyses, while those who had discontinued the *EGFR* TKI for personal reasons or side effects were excluded. The objective treatment response was assessed according to the Response Evaluation Criteria in Solid Tumors (RECIST) version 1.1. A computed tomography of the chest was obtained three months after the initiation of a *EGFR* TKI to determine the 3-month treatment response. To determine the progression-free survival, these patients were followed till either disease progression or 16 January 2019.

### 2.4. Statistical Analysis

The baseline characteristics, including sex, age, performance status, tumor stage, primary tumor size, lymph node involvement, distant metastases, and *EGFR* mutation in tissue sample, were compared between the concordant and discordant groups. Categorical and continuous variables were analyzed using Chi-square test and Student’s *t*-test, respectively. The effects of factors in predicting concordant *EGFR* mutation test results in liquid/tissue biopsy were assessed using logistic regression analysis. The odds ratio (OR) with 95% confidence interval (CI) was reported. Following univariate analyses, all factors were included to build a maximal model of multivariable analysis. The reduced multivariable model was then developed with backward variable selection method, keeping only variables with *p* value less than 0.1, from the maximal model.

In the outcome analyses, which included only stage IV patients receiving first-line *EGFR* TKIs, the initial objective response rate (ORR) and disease control rate (DCR) were calculated. The PFS of patients in concordant and discordant groups were assessed with the Kaplan–Meier method and compared with log-rank test. The effects of factors in predicting PFS were assessed using Cox regression analysis. The hazard ratio (HR) with 95% CI was reported. Following univariate analyses, all factors were included to build maximal models of multivariable analyses. The reduced multivariable models were then developed with backward variable selection method, keeping only variables with *p* value less than 0.1, from the maximal models.

The statistical analyses were performed using SAS system (version 9.4 for Windows, SAS Institute Inc., Cary, NC, USA). A two-sided *p* value of <0.05 was taken as the statistical significance level.

## 3. Results

### 3.1. Predicting Factors for Concordant EGFR Mutation Detected in Liquid/Tissue Biopsy

**Figure 1:**
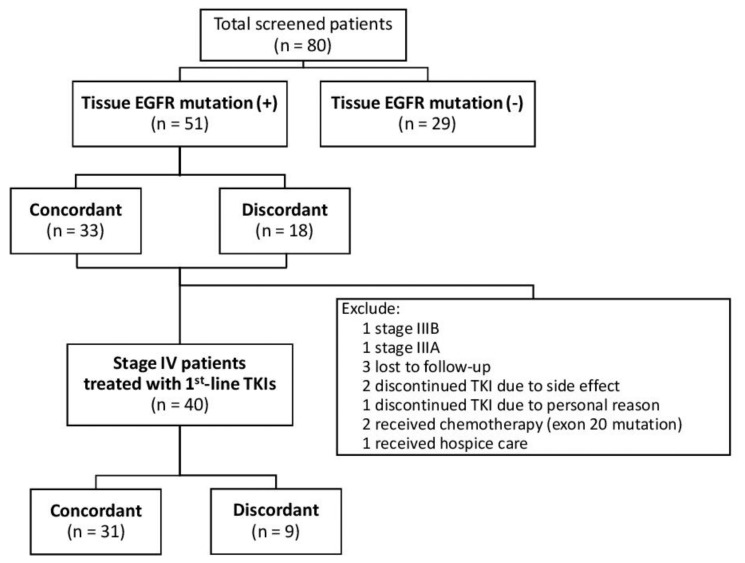
Flow diagram of eligible study population. From the 80 screened patients, 51 patients had detectable *EGFR* mutation in their tissue samples. From these 51 patients, 40 patients who received treatment with a first-line *EGFR* TKI were enrolled in the outcome analysis. Abbreviations: EGFR = epidermal growth factor receptor; TKI = tyrosine kinase inhibitor.

We identified 80 treatment-naïve stage III or IV lung adenocarcinoma patients during the study period, and 51 (63.75%) patients had *EGFR* mutation detected in their tumor tissue samples (Figure 1). These patients had a mean (±standard deviation) age of 64.2 (±10.5) years, and 17 (33.3%) patients were male. Two patients had stage IIIA cancer, one patient had stage IIIB cancer, and the remaining 48 (94%) patients had stage IV disease.

In the 51 patients with *EGFR* mutation detected in the tumor tissue, 33 (65%) patients had concordant *EGFR* mutation test results in liquid biopsy, while 18 (35%) patients had discordant results (Figure 1, Table 1). The patients with concordant results had similar sex distribution, age, and performance status as those with discordant results. The patients with concordant results had significantly higher rate of lymph node involvement (N1–3 disease) than those with discordant results (82% vs. 39%, *p* = 0.0019). The patients with concordant results were all stage IV, whereas only 83% of those with discordant results were stage IV (*p* = 0.0156). The patients with concordant results, compared with those having discordant results, had significantly higher rates of metastases to brain (36% vs. 6%, *p* = 0.0158) and bone (61% vs. 17%, *p* = 0.0026). No patient with concordant results had mutation in exon 20, while 5 (28%) patients in the discordant group had exon 20 mutation (*p* = 0.0014).

Using univariate logistic regression analysis, we found a few predicting factors for concordant *EGFR* mutation test results in liquid/tissue biopsy, including lymph node involvement (N1–3), brain metastasis, and bone metastasis (Table 2). In the reduced model of multivariable analysis, which was developed with backward variable selection method, only lymph node involvement (adjusted OR (95% CI): 8.71 (1.88–40.35), *p* = 0.0057) and bone metastasis (adjusted OR (95% CI): 9.65 (1.72–54.05), *p* = 0.0099) remained the independent predicting factors for concordant *EGFR* mutation test results in liquid/tissue biopsy.

### 3.2. Predicting Factors for PFS in Stage IV Patients Receiving EGFR TKI

From the 51 patients (Figure 1), 40 patients with stage IV lung adenocarcinoma treated with an *EGFR* TKI as their first-line therapy were enrolled in the following outcome analysis (Table 3). These patients included 31 (78%) patients with concordant *EGFR* mutation detected in liquid/tissue biopsy and nine (23%) patients with discordant results (Table 3). The patients with concordant results had similar sex, age, and performance status as those with discordant results. The patients with concordant results had significantly higher rate of lymph node involvement (N1–3 disease) than those with discordant results (84% vs. 33%, *p* = 0.0028). The *EGFR* mutation sites did not differ significantly between two groups and no patient in either group had exon 20 mutation. In terms of the first-line *EGFR* TKI used, 18 (58%) patients in the concordant group took erlotinib, while 6 (67%) of patients in the discordant group took afatinib (*p* = 0.0243).

**Figure 2:**
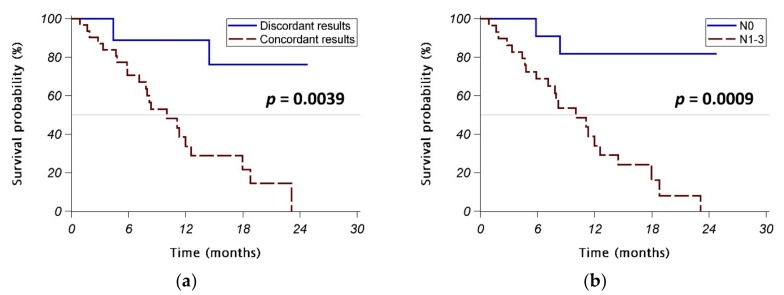
Progression-free survival (PFS) in stage IV patients receiving first-line *EGFR* TKIs. (**a**) Patients with concordant *EGFR* mutation test results in liquid/tissue biopsy vs. those with discordant results. (**b**) Patients with lymph node involvement (N1–3 disease) vs. those without lymph node involvement (N0 disease). Abbreviations: EGFR = epidermal growth factor receptor; ECOG = Eastern Cooperative Oncology Group; TKI = tyrosine kinase inhibitors.

The initial objective response rate was similar in the concordant group and the discordant group (58% vs. 56%, *p* = 0.8934), as was the disease control rate (87% vs. 100%, *p* = 0.2560). However, the patients with concordant *EGFR* mutation test results in liquid/tissue biopsy had a significantly poorer PFS than those with discordant results (*p* = 0.0039) (Figure 2a). Interestingly, patients with lymph node involvement (N1–3 disease) also had significantly poorer PFS than those without lymph node involvement (N0 disease) (*p* = 0.0009) (Figure 2b).

Univariate Cox regression analyses identified factors significantly associated with poorer PFS included concordant *EGFR* mutation detected in liquid/tissue biopsy, lymph node involvement, brain metastasis, and bone metastasis (Table 4). Three *EGFR* TKIs showed similar effects in these patients. Multivariable analyses revealed that lymph node involvement was the only independent prognostic factor for poorer PFS (adjusted HR (95% CI): 7.53 (1.70–33.26), *p* = 0.0078 in reduced model 1 and 8.34 (1.92–36.22), *p* = 0.0047 in reduced model 2).

### 3.3. Sensitivity Analyses

To eliminate the potential bias introduced by different *EGFR* mutation test kits used for tissue specimens, we performed another set of analyses (sensitivity analyses). The six patients using Cobas^®^ *EGFR* Mutation Test v2 for their tissue samples were excluded (Table A1). In consistence with our previous findings, univariate logistic regression analysis showed the same predicting factors for concordant *EGFR* mutation test results in liquid/tissue biopsy, including lymph node involvement (N1–3), brain metastasis, and bone metastasis (Table A2). In the reduced model of multivariable analysis, developed with the backward variable selection method, only lymph node involvement (adjusted OR (95% CI): 9.90 (1.91–51.37), *p* = 0.0064) and bone metastasis (adjusted OR (95% CI): 9.91 (1.62–60.61), *p* = 0.0131) remained the independent predicting factors for concordant *EGFR* mutation test results in liquid/tissue biopsy (Table A3).

**Figure A1:**
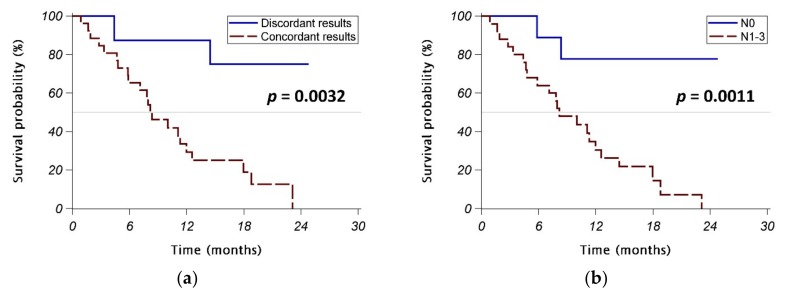
Progression-free survival (PFS) in stage IV patients receiving first-line *EGFR* TKIs (only patients using Qiagen^®^ *EGFR* RGQ PCR kit for their tissue samples). (**a**) Patients with concordant *EGFR* mutation test results in liquid/tissue biopsy vs. those with discordant results. (**b**) Patients with lymph node involvement (N1–3 disease) vs. those without lymph node involvement (N0 disease). Abbreviations: EGFR = epidermal growth factor receptor; ECOG = Eastern Cooperative Oncology Group; TKI = tyrosine kinase inhibitors.

In the 34 patients with stage IV lung adenocarcinoma treated with a first-line *EGFR* TKI (Table A3), those with concordant *EGFR* mutation test results in liquid/tissue biopsy had significantly poorer PFS than those with discordant results (*p* = 0.0032) (Figure A1a). Similarly, patients with lymph node involvement (N1–3 disease) also had significantly poorer PFS than those without lymph node involvement (N0 disease) (*p* = 0.0011) (Figure A1b).

## 4. Discussion

Determining *EGFR* mutation status is important in guiding the treatment for advanced lung adenocarcinoma. Liquid biopsy, which uses plasma ctDNA as surrogates for tissue samples, has been increasingly used in clinical practice. However, it remains unclear whether plasma ctDNA provides the same information about EGFR mutation status as do tissue samples. In the current study, we found that 65% of patients with newly diagnosed advanced lung adenocarcinoma harboring *EGFR* mutation in their tissue samples had concordant *EGFR* mutation testing results in the testing using liquid biopsy. The factors independently associated with the concordant results in liquid/tissue biopsy included lymph node involvement and bone metastasis. We further showed that the concordant results in liquid/tissue biopsy was associated with significantly poorer PFS in stage IV patients treated with *EGFR* TKIs. However, multivariable analysis showed that only lymph node involvement was the independent predicting factor for poorer PFS, while the concordant results in liquid/tissue biopsy was not an independently predicting factor.

There are several advantages of using liquid biopsy to determine the tumor genotypes. It is less invasive than traditional tissue biopsy, so the risk of biopsy-related complications can be eliminated. Liquid biopsy may provide similar results of *EGFR* testing as tissue biopsy. In a recent study analyzing the association between plasma genotyping and treatment outcomes of osimertinib in advanced NSCLC patients who failed to the first-line *EGFR* TKIs therapy, patients with T790M mutation detected by either liquid biopsy or tissue biopsy had similar outcomes [27]. In addition, liquid biopsy provides the results of tumor genotypes more rapidly than traditional tissue biopsy. A recent study revealed that the median turnaround time for *EGFR* gene analysis in newly diagnosed lung cancer patients was three business days while using liquid biopsy and was twelve business days while using tissue biopsy [13]. Finally, investigating plasma ctDNA from cancer patients can account for molecular heterogeneity, because ctDNA fragments from all parts of cancer tissues throughout the patient’s body are collected [28,29,30,31]. In our study, two patients had different *EGFR* genotypes shown in tumor tissue and ctDNA, which might be related to molecular heterogeneity.

The association between clinical features and detectable ctDNA has been investigated in some previous studies. Although a few studies found no correlation between ctDNA levels and tumor burden [32,33], further studies still suggested that the presence of ctDNA was significantly associated with a larger tumor burden [34,35,36,37]. A recent study of late-staged NSCLC patients even showed that patients with bone metastasis had significantly higher ctDNA quantities than those without bone metastases [38]. A recent Korean study of 57 patients with adenocarcinoma harboring activating *EGFR* mutations found that bone metastasis was the only independent factor predicting ctDNA detection [39]. Similar to their findings, our current study found that the concordant *EGFR* testing results in liquid/tissue biopsy was associated with lymph node involvement, brain metastasis, and bone metastasis. Multivariable analysis showed that lymph node involvement and bone metastasis were independent predicting factors for the concordant results in liquid/tissue biopsy, while a trend of association between larger original tumor burden (T3–4) and the concordant results was also noted. Based on our findings, liquid biopsy, rather than repeated tissue biopsies, might be considered first for patients with lung adenocarcinoma with lymph node involvement and/or bone metastasis, especially for patients with high risk of biopsy-related complications. Our findings also suggested that the concordant results in liquid/tissue biopsy might be related to a more extensive tumor burden.

The detectable ctDNA might suggest poorer clinical outcomes because circulating mutant DNA has been found quite useful in assessing tumor dynamics [40]. In the BENEFIT study, a multicenter, single-arm, phase 2 clinical trial in 15 centers in China, patients with clearance of *EGFR* mutations in ctDNA at week 8 had longer PFS than those whose *EGFR* mutations persisted at week 8 [41]. The recent Korean study found that ctDNA detection was associated with poorer PFS in patients treated with *EGFR* TKIs, and also identified ctDNA detection and extrathoracic lymph node metastasis as independent factors predicting poorer PFS [39]. Similar to their findings, our current study showed that concordant *EGFR* testing results in liquid/tissue biopsy was significantly associated with a poor PFS. In contrast to their findings, the predicting effect of concordant EGFR testing results in liquid/tissue biopsy became insignificant after adjusting with other variables, especially lymph node involvement. Our finding suggested that lymph node involvement might be a confounding factor intervening between concordant results in liquid/tissue biopsy and poorer PFS. Our study was different from the Korean study in several aspects: Firstly, we included only stage IV patients actually receiving EGFR TKIs in the outcome analysis, whereas the Korean study included some patients with earlier stage (M0), which might bias the analysis. Secondly, the TKI used was included in the analysis of our study, whereas the Korean study did not include TKI used in their analysis because almost all of their patients used gefitinib (55 patients used gefitinib, one patient used erlotinib, and one patient used afatinib). Thirdly, we included lymph node involvement in the analysis and found it was independently associated with poorer PFS.

Our study still has some limitations. Firstly, the number of enrolled patients was relatively small. Nevertheless, our study was one of the largest studies discussing this topic. Currently, liquid biopsy to detect *EGFR* mutation is usually performed on the failure of first-line *EGFR* TKI in clinical practice, so not many patients received liquid biopsy before starting their first-line treatment. Secondly, the follow-up time was relatively short, so overall survival could not be assessed. Further follow-up study is needed to investigate the association between concordant *EGFR* testing results in liquid/tissue biopsy and overall survival. Finally, our study adopted different approaches to tissue analysis (Qiagen^®^ *EGFR* RGQ PCR kit or Cobas^®^ *EGFR* Mutation Test v2) and plasma ctDNA analysis (Cobas^®^ *EGFR* Mutation Test v2). The two approaches might have different detection limits. Due to low concentrations of ctDNA in plasma, the detection rate of gene mutation by liquid biopsy might be relatively lower. This might affect the results of our study. However, the tests have been adopted in many previous studies [21,22,23,24,25,26,27,42], and previous validation examination in our hospital showed excellent correlation between the results obtained from these two kits. We also performed sensitivity analysis, i.e., another set of analysis excluding those using Cobas^®^ *EGFR* Mutation Test v2 for their tissue samples and found consistent results. Furthermore, the aim of this study is to identify the predicting factors for concordant results from both tests. All patients in the sensitivity analyses had their tumor tissue examined with the Qiagen^®^ kit and their plasma examined with the Cobas^®^ kit. We therefore believe that using different analyzing methods for different sample types might minimally affect the results of this study. 

## 5. Conclusions

Based on our findings, in advanced lung adenocarcinoma patients having insufficient tissue samples for *EGFR* mutation testing, liquid biopsy to determine *EGFR* mutation status might be particularly useful if they have lymph node involvement and/or bone metastasis. We also demonstrated that the concordant results in liquid/tissue samples might indicate a larger tumor burden, as evidenced by the presence of lymph involvement, which actually contributes to poorer PFS. Physicians should be cautious in interpreting results of cell-free DNA assay.

## Figures and Tables

**Table 1:** Baseline characteristics of the study population, stage III or IV lung adenocarcinoma patients having *EGFR* mutations detected in the tissue samples.

Characteristics	*EGFR* Mutation Test Results in Liquid/Tissue Biopsy		*p* Value
	Concordant (*n* = 33)	Discordant (*n* = 18)	
Sex			0.5342
Female	21 (64%)	13 (72%)	
Male	12 (36%)	5 (28%)	
Age (year)	63.2 ± 10.2	66.2 ± 11.0	0.3358
Age:			0.9448
<65 years	15 (45%)	8 (44%)	
≥65 years	18 (55%)	10 (56%)	
Performance status:			0.2412
ECOG 0–1	32 (97%)	16 (89%)	
ECOG 2–4	1 (3%)	2 (11%)	
Advanced primary tumor (T3–4)	27 (82%)	12 (67%)	0.2228
Lymph node involvement (N1–3)	27 (82%)	7 (39%)	0.0019
Metastasis (M1)	33 (100%)	15 (83%)	0.0156
Metastasis to:			
Brain	12 (36%)	1 (6%)	0.0158
Lung	12 (36%)	8 (44%)	0.5722
Bone	20 (61%)	3 (17%)	0.0026
Pleural space	18 (55%)	9 (50%)	0.7560
Liver	5 (15%)	1 (6%)	0.3094
Pericardial space	7 (21%)	1 (6%)	0.1418
Adrenal gland	5 (15%)	1 (6%)	0.3094
Other site	4 (12%)	0 (0%)	0.1239
Mutation site:			
Exon 18	1 (3%)	1 (6%)	0.6571
Exon 19	17 (52%)	7 (39%)	0.3880
Exon 20	0 (0%)	5 (28%)	0.0014
Exon 21	15 (45%)	6 (33%)	0.4006

Data were presented as mean ± standard deviation or number (percentage). Abbreviations: EGFR = epidermal growth factor receptor; ECOG = Eastern Cooperative Oncology Group.

**Table 2:** Predicting factors for concordant *EGFR* mutation detected in liquid/tissue biopsy.

Variables	Univariate Analysis		Multivariable Analysis—Maximal Model		Multivariable Analysis—Reduced Model ^†^	
	OR (95% CI)	*p* Value	OR (95% CI)	*p* Value	OR (95% CI)	*p* Value
Male (vs. female)	1.49 (0.42–5.19)	0.5354	1.83 (0.28–12.11)	0.5296		
Age ≥65 (vs. <65)	0.96 (0.30–3.05)	0.9448	1.46 (0.19–11.27)	0.7161		
ECOG ≥2 (vs. ≤1)	0.25 (0.02–2.97)	0.2722	0.06 (0.00–21.15)	0.3423		
T3–4 (vs. T1–2)	2.25 (0.60–8.42)	0.2286	10.48 (0.87–126.59)	0.0645	5.45 (0.98–30.24)	0.0527
N1–3 (vs. N0)	7.07 (1.93–25.85)	0.0031	10.06 (1.28–78.79)	0.0280	8.71 (1.88–40.35)	0.0057
Brain metastasis (with vs. without)	9.71 (1.15–82.39)	0.0371	5.87 (0.36–96.65)	0.2153		
Lung metastasis (with vs. without)	0.71 (0.22–2.30)	0.5728	0.31 (0.04–2.47)	0.2666		
Bone metastasis (with vs. without)	7.69 (1.85–31.91)	0.0049	8.15 (0.99–66.95)	0.0510	9.65 (1.72–54.05)	0.0099
Pleural metastasis (with vs. without)	1.20 (0.38–3.79)	0.7561	0.49 (0.06–3.80)	0.4954		
Liver metastasis (with vs. without)	3.04 (0.33–28.23)	0.3291	5.26 (0.29–96.02)	0.2630		
Pericardial metastasis (with vs. without)	4.57 (0.52–40.56)	0.1721	0.99 (0.04–22.00)	0.9943		
Adrenal metastasis (with vs. without)	3.04 (0.33–28.23)	0.3291	3.85 (0.19–78.24)	0.3808		
Metastasis to other sites (with vs. without)	^‡^		^‡^			

Abbreviations: EGFR = epidermal growth factor receptor; OR = odds ratio; CI = confidence interval; ECOG = Eastern Cooperative Oncology Group. ^†^ Reduced multivariable models were developed with backward variable selection method, keeping only variables with *p* value less than 0.1, from the maximal model. ^‡^ Because all cases with metastasis to other sites were in the concordant group, odds ratio could not be estimated. This variable was therefore not included in the models.

**Table 3:** Characteristics of the stage IV patients receiving first-line *EGFR* TKIs.

Characteristics	*EGFR* Mutation Test Results in Liquid/Tissue Biopsy		*p* Value
	Concordant (*n* = 31)	Discordant (*n* = 9)	
Sex			0.4546
Female	20 (65%)	7 (78%)	
Male	11 (35%)	2 (22%)	
Age (year)	63.4 ± 10	63.0 ± 9.9	0.9192
Age:			0.5825
<65 years	14 (45%)	5 (56%)	
≥65 years	17 (55%)	4 (44%)	
Performance status:			0.3393
ECOG 0–1	30 (97%)	8 (89%)	
ECOG 2–4	1 (3%)	1 (11%)	
Advanced primary tumor (T3–4)	25 (81%)	5 (56%)	0.1260
Lymph node involvement (N1–3)	26 (84%)	3 (33%)	0.0028
Metastasis (M1)	31 (100%)	9 (100%)	0.0401
Metastasis to:			
Brain	11 (35%)	0 (0%)	0.0358
Lung	11 (35%)	5 (56%)	0.2792
Bone	19 (61%)	2 (22%)	0.0388
Pleural space	17 (55%)	6 (67%)	0.5274
Liver	5 (16%)	1 (11%)	0.7105
Pericardial space	7 (23%)	0 (0%)	0.1165
Adrenal gland	5 (16%)	1 (11%)	0.7105
Other site	4 (13%)	0 (0%)	0.2560
Mutation site:			
Exon 18	1 (3%)	0 (0%)	0.5853
Exon 19	16 (52%)	6 (67%)	0.4242
Exon 21	14 (45%)	3 (33%)	0.5274
TKI used:			0.0243
Gefitinib	6 (19%)	2 (22%)	
Erlotinib	18 (58%)	1 (11%)	
Afatinib	7 (23%)	6 (67%)	
Initial treatment response:			0.4320
Partial response (PR)	18 (58%)	5 (56%)	
Stable disease (SD)	9 (29%)	4 (44%)	
Progressive disease (PD)	4 (13%)	0 (0%)	
Objective response rate	18 (58%)	5 (56%)	0.8934
Disease control rate	27 (87%)	9 (100%)	0.2560

Data were presented as mean ± standard deviation or number (percentage). Abbreviations: EGFR = epidermal growth factor receptor; ECOG = Eastern Cooperative Oncology Group; TKI = tyrosine kinase inhibitors.

**Table 4:** Predicting factors for progression-free survival (PFS) in stage IV patients receiving first-line *EGFR* TKIs.

Variable	Univariate Analysis		Multivariable Analysis—Maximal Model 1		Multivariable Analysis—Reduced Model 1 ^†^		Multivariable Analysis—Maximal Model 2		Multivariable Analysis—Reduced Model 2 ^†^	
	HR (95% CI)	*p* Value	HR (95% CI)	*p* Value	HR (95% CI)	*p* Value	HR (95% CI)	*p* Value	HR (95% CI)	*p* Value
Concordant (vs. discordant) ^‡^	6.71 (1.54–29.23)	0.0112	4.70 (0.32–69.53)	0.2604			5.11 (0.69–37.59)	0.1092		
Male (vs. female)	1.34 (0.59–3.04)	0.4849	0.85 (0.26–2.76)	0.7890			1.55 (0.56–4.28)	0.3969		
Age ≥65 (vs. <65)	1.22 (0.54–2.73)	0.6337	2.69 (0.53–13.72)	0.2349			1.81 (0.62–5.25)	0.2752		
ECOG ≥ 2 (vs. ≤1)	0.75 (0.10–5.59)	0.7783	1.02 (0.08–13.35)	0.9858			0.98 (0.11–8.65)	0.9826		
T3–4 (vs. T1–2)	2.11 (0.71–6.22)	0.1780	1.11 (0.18–6.90)	0.9086			0.91 (0.24–3.43)	0.8950		
N1–3 (vs. N0)	8.34 (1.92–36.22)	0.0047	6.42 (1.09–37.92)	0.0401	7.53 (1.70–33.26)	0.0078	4.35 (0.80–23.49)	0.0879	8.34 (1.92–36.22)	0.0047
Number of metastatic site(s) ≥2 (vs. ≤1)	2.46 (0.97–6.27)	0.0585					3.23 (1.03–10.14)	0.0447		
Metastasis to: (with vs. without)										
Brain	3.12 (1.32–7.35)	0.0094	1.75 (0.43–7.08)	0.4351						
Lung	0.96 (0.42–2.19)	0.9314	0.57 (0.10–3.07)	0.5098						
Bone	2.76 (1.15–6.65)	0.0234	2.57 (0.84–7.92)	0.0994	2.26 (0.92–5.58)	0.0766				
Pleural space	1.59 (0.67–3.75)	0.2911	3.09 (0.83–11.6)	0.0938						
Liver	0.77 (0.26–2.26)	0.6308	0.35 (0.06–2.02)	0.2414						
Pericardial space	1.55 (0.57–4.18)	0.3913	0.72 (0.14–3.63)	0.6877						
Adrenal gland	1.42 (0.42–4.82)	0.5712	2.20 (0.29–16.54)	0.4436						
Other site	1.59 (0.46–5.55)	0.4637	0.48 (0.06–3.68)	0.4776						
TKI used:										
Gefitinib	ref		ref				ref			
Erlotinib	1.07 (0.38–3.04)	0.8927	1.54 (0.25–9.62)	0.6413			0.60 (0.17–2.12)	0.4254		
Afatinib	0.75 (0.23–2.48)	0.6429	1.79 (0.27–11.96)	0.5473			1.28 (0.30–5.43)	0.7344		

Abbreviation: HR = hazard ratio; CI = confidence interval; *EGFR* = epithelial growth factor receptor; TKI = tyrosine kinase inhibitors. ^†^ Reduced multivariable models were developed with backward variable selection method, keeping only variables with *p* value less than 0.1, from the maximal model. ^‡^ Concordant vs. discordant *EGFR* mutation test results in tissue and liquid biopsies.

## Appendix A

**Table A1:** Baseline characteristics of the study population, stage III or IV lung adenocarcinoma patients having *EGFR* mutations detected in the tissue samples (only patients using Qiagen^®^ *EGFR* RGQ PCR kit for their tissue samples).

Characteristics	*EGFR* Mutation Test Results in Liquid/Tissue Biopsy		*p* Value
	Concordant (*n* = 28)	Discordant (*n* = 17)	
Sex			0.3671
Female	16 (57%)	12 (71%)	
Male	12 (43%)	5 (29%)	
Age (year)	64 ± 10.4	66.6 ± 11.2	0.4348
Age:			0.9119
<65 years	12 (43%)	7 (41%)	
≥65 years	16 (57%)	10 (59%)	
Performance status:			0.2854
ECOG 0–1	27 (96%)	15 (88%)	
ECOG 2–4	1 (4%)	2 (12%)	
Advanced primary tumor (T3–4)	22 (79%)	11 (65%)	0.3078
Lymph node involvement (N1–3)	23 (82%)	7 (41%)	0.0047
Metastasis (M1)	28 (100%)	14 (82%)	0.0214
Metastasis to:			
Brain	10 (36%)	1 (6%)	0.0240
Lung	10 (36%)	7 (41%)	0.7141
Bone	16 (57%)	3 (18%)	0.0093
Pleural space	16 (57%)	8 (47%)	0.5109
Liver	5 (18%)	1 (6%)	0.2519
Pericardial space	5 (18%)	1 (6%)	0.2519
Adrenal gland	4 (14%)	1 (6%)	0.3845
Other site	3 (11%)	0 (0%)	0.1624
Mutation site:			
Exon 18	1 (4%)	1 (6%)	0.7153
Exon 19	14 (50%)	7 (41%)	0.5651
Exon 20	0 (0%)	5 (29%)	0.0023
Exon 21	13 (46%)	5 (29%)	0.2586

Data were presented as mean ± standard deviation or number (percentage). Abbreviations: EGFR = epidermal growth factor receptor; ECOG = Eastern Cooperative Oncology Group.

**Table A2:** Predicting factors for concordant *EGFR* mutation detected in liquid/tissue biopsy (only patients using Qiagen^®^ *EGFR* RGQ PCR kit for their tissue samples).

Variables	Univariate Analysis		Multivariable Analysis—Maximal Model		Multivariable Analysis—Reduced Model ^†^	
	OR (95% CI)	*p* Value	OR (95% CI)	*p* Value	OR (95% CI)	*p* Value
Male (vs. female)	1.80 (0.50–6.50)	0.3697	1.99 (0.29–13.58)	0.4828		
Age ≥65 (vs. <65)	0.93 (0.28–3.17)	0.9120	1.41 (0.17–11.93)	0.7537		
ECOG ≥2 (vs. ≤1)	0.28 (0.02–3.32)	0.3118	0.06 (0.00–26.27)	0.3675		
T3–4 (vs. T1–2)	2.00 (0.52–7.66)	0.3118	10.10 (0.75–136.82)	0.0820	4.95 (0.87–28.27)	0.0720
N1–3 (vs. N0)	6.57 (1.68–25.78)	0.0069	12.91 (1.28–130.23)	0.0301	9.90 (1.91–51.37)	0.0064
Brain metastasis (with vs. without)	8.89 (1.02–77.32)	0.0477	2.43 (0.15–38.28)	0.5288		
Lung metastasis (with vs. without)	0.79 (0.23–2.73)	0.7143	0.27 (0.03–2.63)	0.2583		
Bone metastasis (with vs. without)	6.22 (1.45–26.64)	0.0138	8.43 (0.92–76.96)	0.0589	9.91 (1.62–60.61)	0.0131
Pleural metastasis (with vs. without)	1.50 (0.45–5.04)	0.5118	0.62 (0.08–4.86)	0.6525		
Liver metastasis (with vs. without)	3.48 (0.37–32.66)	0.2754	5.49 (0.30–101.12)	0.2520		
Pericardial metastasis (with vs. without)	3.48 (0.37–32.66)	0.2754	1.01 (0.03–29.49)	0.9965		
Adrenal metastasis (with vs. without)	2.67 (0.27–26.09)	0.3993	4.61 (0.19–114.06)	0.3507		
Metastasis to other site (with vs. without)	^‡^		^‡^			

Abbreviations: EGFR = epidermal growth factor receptor; OR = odds ratio; CI = confidence interval; ECOG = Eastern Cooperative Oncology Group. ^†^ Reduced multivariable models were developed with the backward variable selection method, keeping only variables with *p* value less than 0.1, from the maximal model. ^‡^ Because all cases with metastasis to other sites were in the concordant group, odds ratio could not be estimated. This variable was therefore not included in the models.

**Table A3:** Characteristics of the stage IV patients receiving first-line *EGFR* TKIs (only patients using Qiagen^®^ *EGFR* RGQ PCR kit for their tissue samples).

Characteristics	*EGFR* Mutation Test Results in Liquid/Tissue Biopsy		*p* Value
	Concordant (*n* = 26)	Discordant (*n* = 8)	
Sex			0.3784
Female	15 (58%)	6 (75%)	
Male	11 (42%)	2 (25%)	
Age (year)	64.3 ± 10.2	63.5 ± 10.5	0.8466
Age:			0.7016
<65 years	11 (42%)	4 (50%)	
≥65 years	15 (58%)	4 (50%)	
Performance status:			0.3630
ECOG 0–1	25 (96%)	7 (88%)	
ECOG 2–4	1 (4%)	1 (13%)	
Advanced primary tumor (T3–4)	20 (77%)	4 (50%)	0.1439
Lymph node involvement (N1–3)	22 (85%)	3 (38%)	0.0083
Metastasis (M1)	26 (100%)	8 (100%)	0.0656
Metastasis to:			
Brain	9 (35%)	0 (0%)	0.0523
Lung	9 (35%)	4 (50%)	0.4336
Bone	15 (58%)	2 (25%)	0.1058
Pleural space	15 (58%)	5 (63%)	0.8091
Liver	5 (19%)	1 (13%)	0.6623
Pericardial space	5 (19%)	0 (0%)	0.1793
Adrenal gland	4 (15%)	1 (13%)	0.8403
Other site	3 (12%)	0 (0%)	0.3143
Mutation site:			
Exon 18	1 (4%)	0 (0%)	0.5734
Exon 19	13 (50%)	6 (75%)	0.2130
Exon 21	12 (46%)	2 (25%)	0.2877
TKI used:			0.0312
Gefitinib	5 (19%)	2 (25%)	
Erlotinib	16 (62%)	1 (13%)	
Afatinib	5 (19%)	5 (63%)	
Initial treatment response:			0.3889
Partial response (PR)	14 (54%)	4 (50%)	
Stable disease (SD)	8 (31%)	4 (50%)	
Progressive disease (PD)	4 (15%)	0 (0%)	
Objective response rate	14 (54%)	4 (50%)	0.8488
Disease control rate	22 (85%)	8 (100%)	0.2376

Data were presented as mean ± standard deviation or number (percentage). Abbreviations: EGFR = epidermal growth factor receptor; ECOG = Eastern Cooperative Oncology Group; TKI = tyrosine kinase inhibitors.
